# miR-411 is up-regulated in FSHD myoblasts and suppresses myogenic factors

**DOI:** 10.1186/1750-1172-8-55

**Published:** 2013-04-05

**Authors:** Naoe Harafuji, Peter Schneiderat, Maggie C Walter, Yi-Wen Chen

**Affiliations:** 1 Center for Genetic Medicine Research, Children’s Research Institute, Washington, DC, USA; 2 Friedrich-Baur-Institute, Department of Neurology, Ludwig-Maximilians-University of Munich, Munich, Germany; 3 Department of Integrative Systems Biology and Department of Pediatrics, George Washington University, Washington, DC, USA; 4 Center for Genetic Medicine Research, Children’s National Medical Center, 111 Michigan Avenue, NW, Washington, DC 20010, USA

**Keywords:** FSHD, microRNA, miR-411, YAF2, YY1, *Myod*, myogenin

## Abstract

**Background:**

Facioscapulohumeral muscular dystrophy (FSHD) is an autosomal dominant muscle disorder, which is linked to the contraction of the D4Z4 array at chromosome 4q35. Recent studies suggest that this shortening of the D4Z4 array leads to aberrant expression of double homeobox protein 4 (DUX4) and causes FSHD. In addition, misregulation of microRNAs (miRNAs) has been reported in muscular dystrophies including FSHD. In this study, we identified a miRNA that is differentially expressed in FSHD myoblasts and investigated its function.

**Methods:**

To identify misregulated miRNAs and their potential targets in FSHD myoblasts, we performed expression profiling of both miRNA and mRNA using TaqMan Human MicroRNA Arrays and Affymetrix Human Genome U133A plus 2.0 microarrays, respectively. In addition, we over-expressed miR-411 in C_2_C_12_ cells to determine the effect of miR-411 on myogenic markers.

**Results:**

Using miRNA and mRNA expression profiling, we identified 8 miRNAs and 1,502 transcripts that were differentially expressed in FSHD myoblasts during cell proliferation. One of the 8 differentially expressed miRNAs, miR-411, was validated by quantitative RT-PCR in both primary (2.1 fold, p<0.01) and immortalized (2.7 fold, p<0.01) myoblasts. In situ hybridization showed cytoplasmic localization of miR-411 in FSHD myoblasts. By analyzing both miRNA and mRNA data using Partek Genomics Suite, we identified 4 mRNAs potentially regulated by miR-411 including YY1 associated factor 2 (YAF2). The down-regulation of YAF2 in immortalized myoblasts was validated by immunoblotting (−3.7 fold, p<0.01). C_2_C_12_ cells were transfected with miR-411 to determine whether miR-411 affects *YAF2* expression in myoblasts. The results showed that over-expression of miR-411 reduced *YAF2* mRNA expression. In addition, expression of myogenic markers including *Myod*, myogenin, and myosin heavy chain 1 (*Myh1*) were suppressed by miR-411.

**Conclusions:**

The study demonstrated that miR-411 was differentially expressed in FSHD myoblasts and may play a role in regulating myogenesis.

## Background

Facioscapulohumeral muscular dystrophy (FSHD) is an autosomal dominant myopathy with estimated prevalence of 1:20,000 [1,2]. The age of onset is often in the second decade of life with nearly complete penetrance (95%) by age 20 [3]. FSHD is characterized by progressive weakness of different muscle groups, often starting with the facial muscles, followed by the shoulder girdle muscles, and moving down to the hip girdle and the extremities [4-6]. Many patients also exhibit a marked left-right asymmetry in muscle involvement [4,7,8]. Patients can have additional symptoms such as severe inflammation in muscles, subclinical hearing loss, and peripheral retinal capillary abnormalities [9-11]. Genetic studies of FSHD have shown that the disease is associated with a deletion of the D4Z4 repeats in the 4q35 subtelomeric region. In individuals without FSHD, this region contains up to 150 copies of the D4Z4 repeats while patients with FSHD only have one to ten copies of the repeat [4,6,12-16].

Each D4Z4 repeat contains a double homeobox protein 4 (*DUX4*) gene. Several *DUX4* splice variants have been reported to be expressed in germ-line cells and myoblasts [17-19]. Although the function of DUX4 is not yet known, the full-length *DUX4* transcript (fl-DUX4) has been shown to be cytotoxic in vivo and ex vivo when ectopically expressed [19-23]. Several studies suggested that p53-dependent cell death plays a major role in the cytotoxicity of DUX4 [23-25]. Recent studies have also shown that a combination of two genomic features is required to cause FSHD. First, the contraction of the D4Z4 repeats causes hypomethylation of the D4Z4 region, allowing *DUX4* mRNA to be transcribed [21,26]. Second, an intact polyadenylation signal in the region distal to the D4Z4 array allows *DUX4* transcripts from the last D4Z4 repeat to be polyadenylated and therefore stable for protein translation. This combination of events leads to the aberrant expression of DUX4 and the downstream molecular changes involved in FSHD [17,21,27]. The aberrant expression of DUX4 in FSHD has been proposed to inhibit myogenesis by suppressing *Myod* regulated pathways and inducing muscle atrophy pathways [20,24,28-33]. However, the regulatory relationship between DUX4 and these pathways is not clear. Currently no effective therapy for FSHD is available. Several pharmacological treatments such as corticosteroids, albuterol, creatine monohydrate, and anti-human myostatin antibody have been tested for their efficacy of treating FSHD, but none showed promising results [34].

MicroRNAs (miRNAs) are short (~22 nucleotides) non-coding RNAs which regulate gene expression by interfering translation or promoting degradation of target mRNAs [35,36]. A mature miRNA is generated through several steps. First, a primary-miRNA (pri-RNA) is transcribed and then cleaved to form a pre-miRNA, which is a single hairpin-shaped stem-loop [37]. Subsequently, the pre-miRNA is exported to the cytoplasm and cleaved into a mature miRNA duplex by Dicer [37,38]. The functional strand is incorporated into the RNA-induced silencing complex (RISC) to form a miRNA-RISC complex [39,40]. In general, the miRNA-RISC complex will cleave the target mRNA when the target sequence is perfectly complementary to the miRNA sequence, or it will interfere with translation of the target mRNA when mismatches are present in the target sequence [40,41]. Many miRNAs are conserved between vertebrates and invertebrates and have been shown to share functions in various cellular processes including embryogenesis, organogenesis, apoptosis, cell cycle regulation and disease development, including muscle disorders [40-43]. Several miRNAs have been shown to play important roles in muscle differentiation, including the miR-1 and miR-133 families, miR-181, miR-214, miR-24, miR-221 and miR-222 [44-51]. In additional to normal muscle growth and maintenance, miRNAs have also been shown to be differentially expressed in disease conditions [52-54]. A global miRNA expression profile of 10 muscle disorders was previously performed and showed that 185 out of the 428 miRNAs examined were differentially expressed in at least one of the 10 different muscle disorders [55]. Among the 185 miRNAs, 62 were up-regulated in FSHD while none was down-regulated. These findings suggest that miRNAs may play a critical role in FSHD, although the mechanisms involved have not been studied. MiR-411 belongs to the miR-379 family and is located in the miR-379/miR-656 cluster within the DLK-DIO3 region on human chromosome 14 [56]. The miR-379/miR-656 cluster is highly conserved in placental mammals [56]. In mouse brain, the expression of the miR-379/miR-656 gene cluster is likely co-regulated by myocyte enhancing factor 2 (Mef2) and is involved in activity-dependent outgrowth of hyppocampal neurons [57]. The function of miR-411 in brain or other tissues is currently unknown. In this study we performed miRNA expression profiling using PCR-based miRNA arrays to identify miRNAs misregulated in FSHD myoblasts. We then used mRNA profiling to identify potential regulatory targets of miR-411, which was significantly upregulated in the miRNA profiling. We further examined the effects of over-expressing miR-411 in C_2_C_12_ myoblasts and its potential role in myogenesis.

## Methods

### Cell culture and immunostaining

Primary myoblasts were obtained from EuroBioBank (Dr. Schneiderat and Dr. Walter) (Additional file 1: Table S1). For expression profiling experiments, cells were cultured in collagen I-coated flasks with SkGM (Lonza) at 37°C, 5% CO_2_. For in situ hybridization experiments, cells were seeded on poly-D-lysine/mouse laminin-coated coverslips (BD BioCoat, BD Biosciences).

C_2_C_12_ cells were purchased from ATCC and cultured in growth medium consisting of DMEM (Life Technologies) with 10% heat-inactivated fetal bovine serum (Sigma). Myotube differentiation was induced by culturing the cells in differentiation medium consisting of DMEM with 2% heat-inactivated horse serum (Sigma) at 37°C, 5% CO_2_.

Immortalized human myoblasts were from the Boston Biomedical Research Institute and cultured as described in previously published protocol [58,59]. Briefly, immortalized myoblasts were cultured in a growth medium consisting of medium 199 and DMEM (Life Technologies) in a 1:4 ratio with 0.8 mM sodium pyruvate (Life Technologies), 3.4 g/l sodium bicarbonate (Sigma-Aldrich), 15% fetal bovine serum (Thermo Scientific), 0.03 μg/ml Zinc sulfate (Fisher), 1.4 μg/ml vitamin B12 (Sigma-Aldrich), 2.5 ng/ml recombinant human hepatocyte growth factor (Millipore), 10 ng/ml basic fibroblast growth factor (BioPioneer), 0.02 M HEPES (Life Technologies), and 0.055 μg/ml dexamethasone (Sigma-Aldrich) at 37°C, 5% CO_2_. The culture dish was coated with 0.1% gelatin (Sigma-Aldrich).

Myoblasts purity was determined by performing immunofluorescent staining using anti-human desmin (Dako) antibody. Myoblasts that exhibited greater than 70% desmin-positive cells were utilized for expression profiling. Immunostaining was conducted as previously described [60]. Briefly, the cells were fixed with 4% paraformaldehyde for 30 minutes, then blocked with 0.3% Triton X-100, 15% hose serum and 450 mM NaCl in phosphate buffer saline (PBS). Following blocking, fixed cells were incubated with anti-human desmin (Dako) for 4°C overnight. After washing 3 times, fixed cells were incubated with secondary antibody, DyLight 488-conjugated donkey anti-mouse IgG (Jackson ImmunoRessearch Laboratories). The slides were mounted using ProLong Gold Antifade Reagent with DAPI (Life Technologies) for further examination.

### Total RNA extraction and miRNA expression profiling

To identify differentially expressed miRNA in FSHD primary myoblasts, we performed miRNA expression profiling using proliferating primary FSHD and control myoblasts (n=3, Additional file 1: Table S1). Total RNA with miRNA enrichment was extracted from cells using *mir*Vana miRNA isolation kit (Life Technologies) according to manufacturer’s protocol. Following RNA isolation, RNA quality and concentration were determined by gel electrophoresis and NanoDrop (Thermo Fisher Scientific), respectively. The miRNA profile of each myoblasts was determined using TaqMan Human MicroRNA Array v2.0 (Human Array A) (Life Technologies) according to manufacturer’s protocol. Briefly, reverse transcription (RT) was performed with 100 ng of total RNA, Multiplex RT Human primer pools, and TaqMan MicroRNA Reverse Transcriptase Kit (Life Technologies). Real-time PCR was performed with TaqMan Universal PCR Master Mix, No AmpErase UNG (Life Technologies) using the Applied Biosystems 7900HT System. Ct values of all miRNAs were determined using RQ Manager 1.2 (Life Technologies) with a threshold of 0.1. Ct values were then imported into Partek Genomics Suite 6.5 and normalized to control, RNU48. ANOVA analysis was performed using Partek Genomics Suite 6.5 (Partek Incorporated, MO). No multiple testing corrections were performed.

### mRNA expression profiling and miRNA target search

The total mRNA samples were expression-profiled using the Affymetrix Human Genome U133A plus 2.0 microarrays (Affymetrix) following the manufacture’s protocol and as previously described [17,61]. Absolute analysis of the array data was performed using the MAS5 algorithm of the Affymetrix expression console. All genes with 10% present calls were then imported into Partek Genomics Suite 6.5 for t-test analysis [62]. Targets of miRNA were predicted using Partek with the TargetScanHuman (release 5.1) database. The identified transcripts were further selected for those down-regulated in the FSHD myoblasts, considering that miRNAs generally down-regulate expression of the target genes.

### Quantitative reverse transcription polymerase chain reaction (qRT-PCR)

Expression levels of miR-411 were analyzed by qRT-PCR using TaqMan MicroRNA assays according to the manufacturer’s instructions. RNU48 served as an endogenous control (Life Technologies). Briefly, RT reactions were performed with 10 ng of total RNA using TaqMan MicroRNA Reverse Transcriptase Kit (Life Technologies). Real-time PCR was performed using TaqMan Universal PCR Master Mix, No AmpErase UNG (Life Technologies) and Applied Biosystems 7900HT System. Relative fold changes were calculated using the comparative C_T_ Method (ΔΔ C_T_ Method) [63,64]. We chose RNU48 as the endogenous control because it demonstrated the most stable expression level among suggested internal control genes noted by Life Technologies.

Expression levels of *Myod*, myogenin, myosin heavy chain 1 (*Myh1*), and YY1 associated factor 2 (*Yaf2*) were determined using qRT-PCR as previously described [65]. 18s rRNA (Life Technologies) was used as the endogenous control. Briefly, RT reaction was performed with random hexamers and Super Script II (Life Technologies). Real-time PCR was performed with SYBR green Master Mix or TaqMan Universal PCR Master Mix (Life Technologies) using a final template concentration of 0.4 ng/μl. *Myod*, myogenin and *Myh1* primers were a gift from Dr. Tatiana V. Cohen [66]. The primer sequences were as follows: *Myod*, 5^′^- GGCTACGACACCGCCTACTA -3^′^; and 5^′^- GCTCCACTATGCTGGACAGG -3^′^. myogenin, 5^′^- GGGCAATGCACTGGAGTT -3^′^; and 5^′^- ATGGTTTCGTCTGGGAAGG -3^′^. *Myh1*, 5^′^- GCAAGAAGCAGATCCAGAAAC -3^′^; and 5^′^- CGGTCTTCCTCAGTTTGATAAG -3^′^. *Yaf2*, 5^′^- ATCAGGGTTAGCGCTGTTGT -3^′^; and 5^′^- TGGCAAGTTCTTTCCTGCTT -3^′^. All the Ct values were extracted with SDS (Life Technologies) and, after normalization using r18s, were converted into expression levels. Student t-tests were performed to determine the statistical significance of changes.

### In situ hybridization

In situ hybridization was performed using 5^′^-digoxigenin (DIG) labeled miRCURY locked nucleic acid (LNA) detection probes, including hsa-miR-411 (38398–01), U6 (99002–01) and Scramble-miR (99004–01) (Exiqon) following the protocol from Exiqon and Roche with few modifications. Cultured cells were fixed with 4% paraformaldehyde (PFA)/PBS, rinsed with 100% methanol, then rehydrated and acetylated. Cells were then pre-hybridized in hybridization buffer at 37°C for 1 hour [67]. The hybridization step was performed with 30 nM heat-denatured LNA-probe at 37°C for overnight. After stringent washes with 50% formamide, 0.1% Tween-20, 2× SSC at 37°C and 0.2× SSC at room temperature, the samples were incubated with blocking solution, and next with alkaline phosphatase (AP)-conjugated anti-DIG Fab fragment (Roche). The antibodies were detected with the 5-bromo 4-chloro-3-indolyl phosphate (BCIP)- nitroblue tetrazolium (NBT) colorimetric detection system incubating the slides in 0.35 mg/ml NBT and 0.175 mg/ml BCIP in 10% polyvinyl alcohol (PVA) and 1 mM levamisole staining solution. The samples were mounted using ProLong Gold Antifade Reagent with DAPI (Life Technologies) for further examination.

### Immunoblotting

Immunoblotting was performed as described previously [25]. Briefly, myoblasts were lysated with RIPA buffer (Sigma-Aldrich) on ice. Protein concentration was determined by DC protein assay (Bio-Rad), and 30 μg of protein was loaded to 12% Bis-Tris NuPAGE Novex gels (Life Technologies) and then transferred to Hybond nitrocellulose membranes (Amersham Biosciences). After blocking, the membrane was incubated with rabbit polyclonal anti-YAF2 antibody (1:2,000) (Aviva) followed by horseradish peroxidase (HRP)-conjugated anti-rabbit antibodies (Amersham Biosciences). Chemiluminescent substrate (Pierce) was used to visualize the target proteins on blue light autorad film (BioExpress). Detection of the loading control, GAPDH (Santa Cruz Biotechnology), was similarly performed with 1:10,000 dilution of the mouse monoclonal primary antibody. The same blot was subsequently incubated with mouse monoclonal anti-human YY1 antibody (1:200) (Bio Matrix Research) to detect the YY1 protein followed by HRP-conjugated anti-mouse antibodies (Amersham Biosciences). Band density of the target protein was measured using a GS 800 Calibrated Densitometer (Bio-Rad) and image J [68] then normalized to the density of GAPDH. Student t-tests were performed to determine the statistical significance of changes. The experiments were performed in quadruplicate.

### Cell Transfection

Cells were transfected with 30 mM Ambion pre-miR-411 precursor oligos (Life Technologies) using Lipofectamine 2000 (Life Technologies) following the manufacture protocol. Briefly, 4×10^3^/cm^2^ C_2_C_12_ cells were plated in growth medium one day before the transfection. Cells were transfected when they reached 70% confluence. A final concentration of 30 nM oligos was used for the transfection. Differentiation was induced 6 hours after the pre-miR-411 transfection.

## Results

### MiRNA-411 was differentially expressed in FSHD primary myoblasts

**Figure 1:**
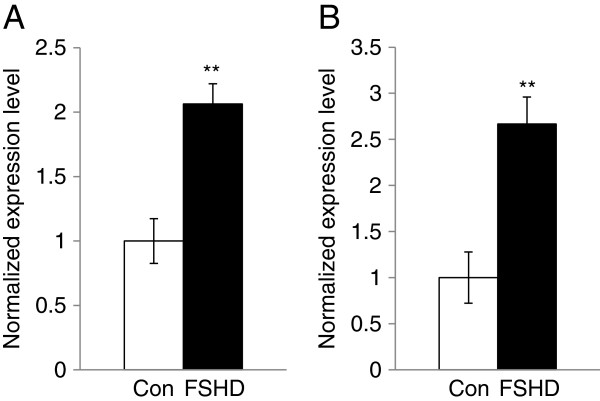
**The up-regulation of miR-411 was validated in primary and immortalized FSHD myoblasts using qRT-PCR.** MiR-411 expression was significantly higher in the proliferating primary myoblasts (**A**) and immortalized myoblasts (**B**) of patients with FSHD (n=4). White columns indicate control (Con) and black columns indicate FSHD myoblasts (FSHD). Error bars represent standard errors, and asterisks (**) indicate p < 0.01.

MiRNAs have been shown to be misregulated in FSHD and other muscular dystrophies and were proposed to be involved in the pathological mechanisms of muscular dystrophies. To identify miRNAs misregulated in proliferating FSHD myoblasts and their potential mRNA targets, we performed miRNA and mRNA expression profiling using total RNA samples isolated from 3 sets of proliferating primary myoblasts. Using TaqMan Human MicroRNA Arrays, we identified 8 miRNAs differentially expressed in FSHD myoblasts comparing to controls (n=3, p<0.05) (Additional file 2: Table S2). To validate the findings, we performed qRT-PCR with an independent set of primary myoblasts (n=4). Only miR-411 was validated using the second set of samples (2.1 fold, p<0.01) (Figure 1A). To determine whether the same difference could be observed in immortalized FSHD myoblasts of which the control myoblasts were derived from an unaffected family member, we performed qRT-PCR and the results showed a similar difference in the immortalized FSHD myoblasts comparing to the control sample (2.7 fold, p<0.01, n=4) (Figure 1B).

**Figure 2:**
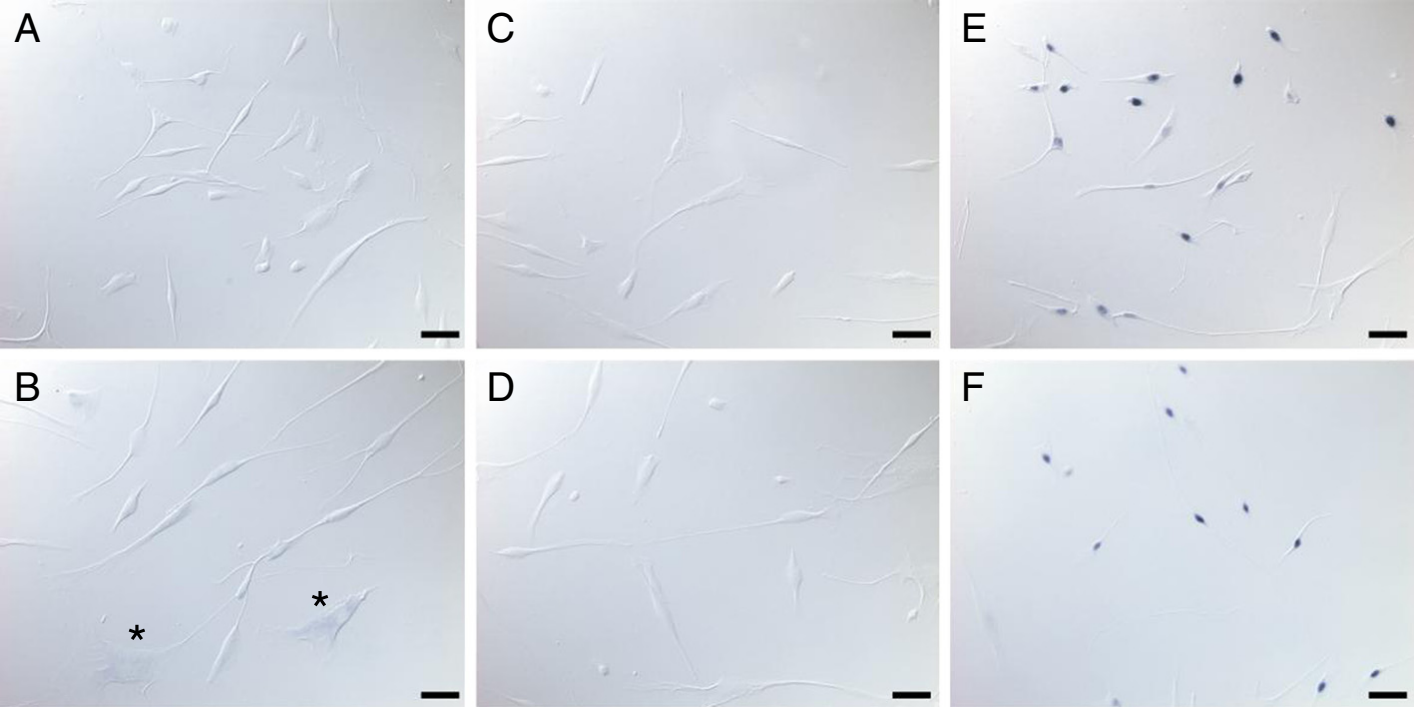
**Cellular localization of miR-411 in FSHD myoblasts.** The cellular localization of miR-411 was determined by in situ hybridization using LNA probes. While not seen in control myoblasts (**A**), the expression of miR-411 was visible in the cytoplasm of some FSHD myoblasts (**B**: marked by asterisks). The negative control using probes with scramble sequences did not generate any signal in control myoblasts (**C**) or FSHD myoblasts (**D**). In contrast, the positive control (U6 probe) showed nuclear staining in both control myoblasts (**E**) and FSHD myoblasts (**F**). The scale bar is 50 μm.

The expression of miR-411 has been reported in brain, however the function and cellular localization of the miRNA is unknown. To determine the cellular localization of the miR-411 in myoblasts, we visualized miR-411 by in situ hybridization. The results showed that miR-411 was localized in the cytoplasm of the myoblasts. A subset of primary FSHD myoblasts showed stronger staining that can be visualized by the in situ hybridization (Figure 2B). The cells which expressed higher level of miR-411 were larger in size. The morphology is more similar to the FSHD cells showing necrotic phenotype reported previously [32,69]. The miR-411 expression in the control myoblasts as well as the negative probes in both the disease and control cells was not visible (Figure 2).

### Over-expression of miR-411 leads to down-regulation of *YAF2*, *MYOD* and myogenin

To identify mRNA transcripts that can be potentially regulated by miR-411, mRNA profiles generated using the same total RNA samples used for miRNA profiling were analyzed to identify potential mRNA targets of miR-411. First, 1,502 mRNA transcripts were differentially expressed in the FSHD myoblasts comparing to the control samples during cell proliferation (p<0.05) (Additional file 3: Table S3). The miR-411 predicted targets were identified then compared to the mRNA transcript list using Partek Genomics Suite 6.5 to identify potential miRNA-mRNA interactions. Among the potential miR-411 targets, we further selected transcripts that were down-regulated in FSHD myoblasts based on the current knowledge that miRNAs negatively regulate target gene expression. Four candidate genes were identified, including *YAF2* (−1.4 fold, p<0.05), calmodulin-like 4 (*CALML4*) (−1.8 fold, p<0.05), mitogen-activated protein kinase kinase 1 (*MAP2K1*) (−1.2 fold, p<0.05), and SH3 and multiple ankyrin repeat domains 2 (*SHANK2*) (−1.9 fold, p<0.05). Among the 4 genes, two genes, YAF2 and MAP2K1, are known to be involved in cell cycle regulation and early myogenesis. In addition, YAF2 negatively regulates myotube differentiation by inhibiting Yin Yang 1 (YY1) activity [70]. YY1 is a transcriptional repressor that inhibits muscle gene expression and myogenesis [71,72]. YAF2 interacts with YY1 and facilitates proteolytic cleavage of YY1 by m-calpain [70].

**Figure 3:**
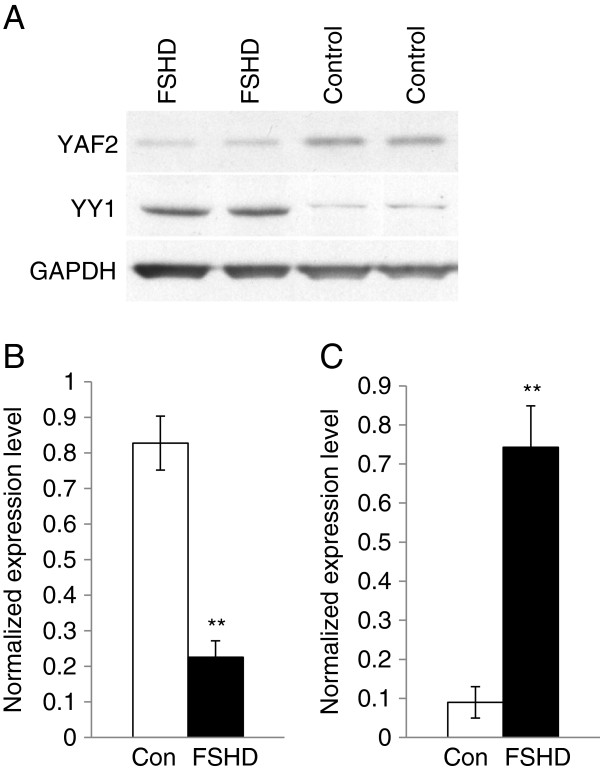
**YAF2 and YY1 proteins were differentially expressed in immortalized FSHD myoblasts.** Immunoblotting of YAF2, YY1 and GAPDH in two sets of the samples (n=4) were shown in (**A**). YAF2 was down-regulated in immortalized FSHD myoblasts compared to control myoblasts (**B**). The YY1 protein was up-regulated in immortalized FSHD myoblasts (**C**). White columns indicate control (Con) and black columns indicate FSHD myoblasts (FSHD). Error bars represent standard errors, and asterisks (**) indicate p < 0.01.

**Figure 4:**
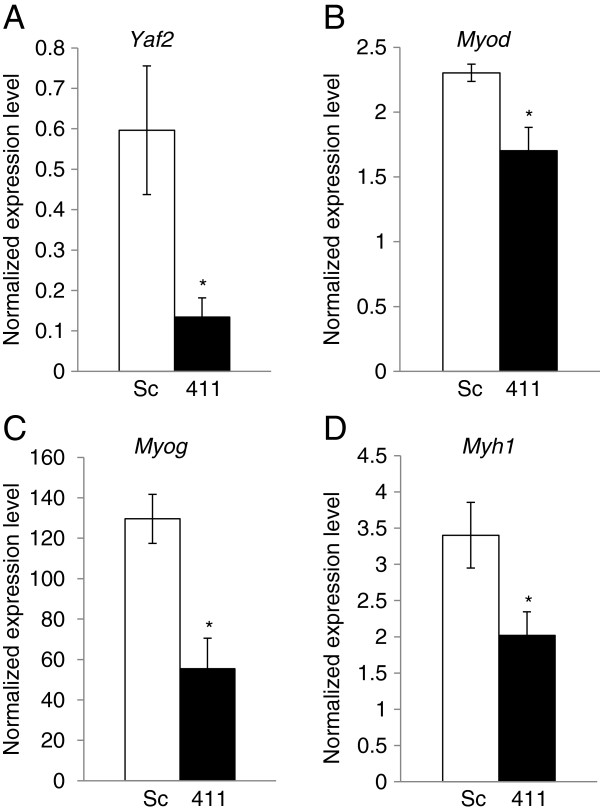
**Over-expression of miR-411 reduced the expression level of differentiation markers in C_2_C_12_ murine myoblasts.** Over-expression of miR-411 down-regulated expression of *Yaf2* (**A**), *Myod* (**B**), myogenin (**C**), and *Myh1* (**D**) as analyzed by real-time qRT-PCR (n=4). Each sample was transfected with miR-411 oligos (411) or negative oligo control (Sc). White columns indicate the control scramble-miRNA oligos (Sc) and black columns indicate the miR-411 oligos (411). Error bars represent standard errors, and an asterisk (*) indicates p < 0.05.

There are five human *YAF2* transcript variants reported (GenBank). All five splice variants have one or two putative miR-411 binding site in their 3^′^ UTR (Additional file 4: Figure S1). Among these, *YAF2* variant 2 has been cloned from the human muscle cDNA library as a protein which interacted with YY1 [70]. To determine if YAF2 expression is affected at the protein level in FSHD myoblasts, we performed immunoblotting and showed a 3.7 fold (p<0.01) down-regulation of YAF2 in FSHD immortalized myoblasts (Figure 3). The expression level of YY1 in the immortalized FHSD myoblasts was determined by immunoblotting. The result showed that YY1 was 8.3 fold (p<0.01) up-regulated in the FSHD myoblasts (Figure 3). To further determine whether miR-411 can regulate YAF2 and downstream pathways, C_2_C_12_ myoblasts were transfected with miR-411 precursor oligos, followed by cell differentiation for 5 days. The expression levels of miR-411, *Yaf2* and myogenic markers, *Myod*, myogenin (*Myog*), and *Myh1* were examined by qRT-PCR. The results showed that *Yaf2* expression level was significantly down-regulated (−4.5 fold, p<0.05) by miR-411 over-expression (Figure 4A). Interestingly, myogenic factors, *Myod* (−1.4 fold, p<0.05) and *Myog* (−2.1 fold, p<0.05) as well as a myotube differentiation marker, *Myh1* (−1.7 fold, p<0.05), were also significantly down-regulated by miR-411 over-expression (Figure 4B-D).

## Discussion

To determine whether the differentially expressed miRNAs identified in our study overlap with changes identified in miRNAs in patient muscle biopsies [55], we compared our miRNA data to the previously published miRNA profiling study of 10 muscle disorders. In the study, expression of 62 out of 428 miRNAs examined was differentially expressed muscles of patients with FSHD. All miRNAs were up-regulated in the muscles while none was down-regulated. One miRNA, miR-517* was reported to be uniquely up-regulated in FSHD. We compared our myoblast miRNA profiling data to the study and found out that 5 out of the 8 miRNAs identified in our study were overlapped with the misregulated miRNAs in the previous study (Additional file 2: Table S2). Among the 5 miRNAs, miR-99b and miR-18a were also reported to be misregulated in FSHD biopsies. However, the directions of expression changes were the opposite in the two studies. While miR-517* was examined in our study, the expression was not detectable in either the FSHD and control myoblasts. The discrepancy may results from differences in sample type (muscle biopsies vs. primary myoblasts), disease stages, or platform used. It is not clear whether a probe detecting miR-411 was on the array used in the previous profiling study therefore it is not known whether miR-411 was changed in patients’ muscle samples. While no study was conducted to investigate the function of miR-411, differential expression of miR-411 was reported in a miRNA profiling study of a mouse model of Duchenne muscular dystrophy known as the *mdx* mouse model [54]. In this study, miR-411 was reported to be down-regulated (−1.8 fold) in the gastrocnimeous muscles of the *mdx* mice. To date, it is not known whether the down-regulation of miR-411 contributes to the disease phenotype or participates in the muscle regeneration process in the *mdx* mouse model. However, our data suggested that reduction of miR-411 may have a positive effect on muscle regeneration considering its potential to suppress factors that promote myoblast maturation.

In this research, we showed that YAF2 was down-regulated in FSHD myoblasts and is a potential target of miR-411. YAF2 is a C_2_C_2_-type zinc finger factor and an inhibitor of the YY1 protein, a known negative regulator of myogenesis [70]. YAF2 shows high similarity with YEAF1 and can be a part of Polycomb group (PcG) complexes [73,74]. YY1 is also a member of the PcG and has been shown to bind to the D4Z4 repeat with HMGB2 and nucleolin [75,76]. In myoblasts, YY1 inhibits differentiation through binding to the promoter regions of myofibrillar genes and the retinoblastoma gene (Rb) which induces terminal exit from the cell cycle [77]. YY1 also inhibits miR-29 which is a positive regulator of myotube differentiation [78]. It has been shown that YY1 binds to the serum response factor (SRF) binding sequence, CArG box, in the regulatory region of the MYOD gene during muscle regeneration, which repressed the expression of MYOD [79]. SRF is a member of the MADS box transcription factor family and a positive regulator of myogenesis [80-83]. It is believed that YY1 inhibits myogenesis via competition for SRF binding sites and repressing expression of myogenic factors, such as MYOD. The expression level of YY1 protein in muscle cells is positively controlled by NF-kappa B which inhibits myogenesis, and negatively regulated by YAF2 [70,84]. *YAF2* expression increases during muscle differentiation and directly binds to YY1 protein. The interaction between YAF2 and YY1 promotes proteolytic cleavage of YY1 by m-calpain, thus reducing YY1 protein levels in differentiating myofibers. Based on the findings, we hypothesized that the over-expression of miR-411 affects the expression of myogenic factors through the inhibition of YAF2 expression. The down-regulation of YAF2 might result in the accumulation of YY1 proteins therefore suppress MYOD and myogenin expression. Previous mRNA profiling studies examining patients’ muscle biopsies showed that YY1 was up-regulated in FSHD biopsies [17]. In addition, YY1 was shown to be up-regulated in immortalized human myoblasts transfected with DUX4 expression vector [85]. In this study, we showed YY1 was up-regulated in FSHD myoblasts. Our findings suggested a potential regulatory mechanism of the YY1 up-regulation and the myogenesis defects observed in FSHD.

While some studies have reported that FSHD myoblasts exhibit a normal, healthy phenotype [28,59,86], others reported an abnormal phenotype regarding their appearance and ability to differentiate [32,33,87]. Our results agree with the latter findings, as we observed some FSHD myoblasts with necrotic features as reported previously [32,69]. Interestingly, these cells also showed visible staining of miR-411 in their cytoplasm, suggesting the up-regulation of miR-411 may be involved at a later stage when the cells are further affected. Despite the phenotypic differences reported in the different groups, several expression profiling studies showed similar defects at the molecular level, such as in cell cycle regulation, early myogenesis, and oxidative stress responses [28,30-32]. MYOD is a master regulator of myogenesis and has been studied extensively [88,89]. MYOD is expressed in the early stages of myogenesis and induces myoblast differentiation through activation of downstream muscle-specific genes including myogenin, *MYH1*, as well as muscle-specific miRNAs, such as miR-1, miR-133 and miR-206 [45,90,91]. MYOD also promotes cell cycle arrest to induce myoblast differentiation through activation of p21 and Rb [92,93]. Overexpression of MYOD has been shown to convert 10T1/2 fibroblasts and other type of cell lines into myoblasts [94,95]. While MYOD is critical in activation of myogenesis, early up-regulation of myogenin, a downstream regulatory target of MYOD, can lead to premature myoblast differentiation, as seen in early myotube formation by FSHD myoblasts [96]. Expression profiling studies have supported this defect in FSHD myoblasts by showing up-regulation of MYOD, myogenin and other MYOD regulated genes in the proliferating cells [29,31,96]. Whether the up-regulation of miR-411 directly contributes to the disease pathogenesis, or is part of a compensatory response to suppress the prematurely activated MYOD program still needs to be further investigated.

## Conclusions

In this study, we demonstrated that miR-411 was up-regulated in both primary and immortalized FSHD myoblasts in comparison to control myoblasts. We also identified YAF2 as a potential regulatory target of miR-411 by analyzing the miRNA and mRNA expression profiling data generated from primary myoblasts. Over-expressing miR-411 in C_2_C_12_ cells leads to down-regulation of *Myod*, myogenin, and *Myh1*. Based on previous findings as well as our own observations, we propose a potential model of how up-regulation of miR-411 can be involved in the myogenic defect observed in FSHD myoblasts. Starting with the up-regulation of miR-411, YAF2 is suppressed, which will then positively regulate YY1. Up-regulation of YY1 suppresses myogenic factors including MYOD and myogenin, which directly affect myogenesis. Alternatively, the up-regulation of miR-411 may be a compensatory mechanism to the hypothesized premature activation of the MYOD program. While each specific regulatory relationship needs to be further investigated, our findings demonstrated a potential role of miR-411 in regulating myogenesis, and provides a novel molecular regulatory mechanism that may be involved in FSHD pathogenesis. In addition, miRNAs circulating in the blood have been acknowledged as readily accessible disease markers. Thus, miR-411 may be a potential candidate as a biomarker in FSHD studies.

## Abbreviations

(FSHD): Facioscapulohumeral muscular dystrophy; (DUX4): double homeobox protein 4; (fl-DUX4): full-length DUX4 transcript; (miRNA): microRNA; (pri-RNA): primary-miRNA; (RISC): RNA-induced silencing complex; (Mef2): myocyte enhancing factor 2; (PBS): phosphate buffer saline; (RT): reverse transcription; (qRT-PCR): quantitative reverse transcription polymerase chain reaction; (Myh1): myosin heavy chain 1; (YAF2): YY1 associated factor 2; (DIG): 5^′^-digoxigenin; (LNA): locked nucleic acid; (PFA): paraformaldehyde; (AP): alkaline phosphatase; (BCIP): 5-bromo 4-chloro-3-indolyl phosphate; (NBT): nitroblue tetrazolium; (PVA): polyvinyl alcohol; (HRP): horseradish peroxidase; (CALML4): calmodulin-like 4; (MAP2K1): mitogen-activated protein kinase kinase 1; (SHANK2): SH3 and multiple ankyrin repeat domains 2; (YY1): Yin Yang 1; (Myog): myogenin; (PcG): Polycomb group; (Rb): retinoblastoma gene; (SRF): serum response factor.

## Competing interests

The authors declare no conflicts of interests.

## Authors’ contributions

PS and MCW provided the primary myoblasts and participated in manuscript preparation. NH performed the experiments and prepared the manuscript. Y-WC designed and supervised the experiments and prepared the manuscript. All authors read and approved the final manuscript.

## Supplementary Material

Additional file 1: Table S1 Human primary myoblasts used for miRNA and mRNA profiling (the top 6 samples) and validation (the bottom 8 samples). Click here for file

Additional file 2: Table S2 miRNAs differentially expressed in proliferating FSHD myoblasts (p<0.05). Click here for file

Additional file 3: Table S3 mRNAs differentially expressed in proliferating FSHD myoblasts (p<0.05). Click here for file

Additional file 4: Figure S1 Putative miR-411 binding sites in the 3^′^ UTR of 5 splice variants of *YAF2*. The seed region of miR-411 is underlined. The complimentary bases are highlighted in yellow. The variant 2 has been reported to be expressed in skeletal muscles. Click here for file
