# Supplementary material for: miR-411 is up-regulated in FSHD myoblasts and suppresses myogenic factors
Source: Orphanet J Rare Dis. 2013 Apr 5;8:55. doi: 10.1186/1750-1172-8-55 (PMC3637251; doi:10.1186/1750-1172-8-55)
Supplement: Additional file 4: Figure S1 — Putative miR-411 binding sites in the 3′ UTR of 5 splice variants of YAF2. The seed region of miR-411 is underlined. The complimentary bases are highlighted in yellow. The variant 2 has been reported to be expressed in skeletal muscles. [file 1750-1172-8-55-S4.docx]

**miR-411** 3’-GCATGCGATATGCCAGATGAT-5’

**Predicted miR-411 binding site**

Variant_1_mRNA (NM_001190979) 5’-TAATTCTTGAATTCTCTACTA-3’

Variant_2_mRNA (NM_005748) 5’-TAATTCTTGAATTCTCTACTA-3’

Variant_3_mRNA (NM_001190977) 5’-TAATTCTTGAATTCTCTACTA-3’

Variant_4_mRNA (NM_001190980) (site 1) 5’-AGTGAGATCTCGTCTCTACTA-3’

(site 2) 5’-CAATCCAGGTGTCTTCTACTA-3’

Variant_5_non-coding (NR_034000) 5’-TAATTCTTGAATTCTCTACTA-3’
